# A20 inhibits osteoclastogenesis via TRAF6‐dependent autophagy in human periodontal ligament cells under hypoxia

**DOI:** 10.1111/cpr.12778

**Published:** 2020-02-06

**Authors:** Ke Yan, Chengyu Wu, Yu Ye, Lu Li, Xiaoqian Wang, Wei He, Shuangshuang Ren, Yan Xu

**Affiliations:** ^1^ Jiangsu Key Laboratory of Oral Diseases Nanjing Medical University Nanjing China; ^2^ Department of Periodontics Affiliated Hospital of Stomatology Nanjing Medical University Nanjing China; ^3^ Department of Neurosurgery Affiliated Aoyang Hospital of Jiangsu University Zhangjiagang China

**Keywords:** autophagy, hypoxia, osteoclast differentiation, periodontal ligament cells, periodontitis, TNFAIP3(A20)

## Abstract

**Objectives:**

A20 exerts an anti‐osteoclastogenic effect through the inhibition of NF‐κB signalling in periodontitis. It also regulates autophagy via ubiquitin modification. This study was aimed at exploring the relationship between A20 and autophagy in anti‐osteoclastogenesis in human periodontal ligament cells (hPDLCs) under hypoxia.

**Materials and Methods:**

Real‐time PCR and Western blot were used to detect relative mRNA and protein levels. The formation of autophagosomes was measured by TEM. Osteoclastic differentiation was assessed by TRAP staining and hydroxyapatite resorption assay. The interactions between different proteins were observed by co‐IP.

**Results:**

Cells cultured under 2% O₂ exhibited decreased A20 expression and increased RANKL/OPG (R/O) ratio. There was a negative correlation between A20 and TRAF6, and similar results were found with autophagic flux. A20 delayed the increase in R/O ratio under hypoxia. Autophagy in hPDLCs and osteoclast differentiation and hydroxyapatite resorption areas in mouse bone marrow mononuclear cells (BMMCs) were inhibited by A20. Moreover, inhibition of autophagy using 3‐MA resulted in increased expression of A20 and decreased number and function of osteoclasts. In addition, A20 inhibited polyubiquitination at K63 and enhanced that at K48 in TRAF6 to suppress autophagy under hypoxic conditions.

**Conclusions:**

A20 inhibits osteoclastogenesis via inhibition of TRAF6‐dependent autophagy in hPDLCs under hypoxia. These findings suggest that A20 may be a key gene target during bone loss in periodontitis via TRAF6‐mediated inhibition of autophagy.

## 1 INTRODUCTION

Periodontitis is a chronic inflammatory disease that can lead to the loss of tooth‐supporting tissue, including the alveolar bone. Receptor activator of nuclear factor‐kappa B ligand (RANKL), receptor activator of nuclear factor‐kappa B (RANK) and osteoprotegerin (OPG) are vital factors that are involved in modulating the differentiation and maturation of osteoclasts.^1^ RANKL promotes the maturation and activation of pre‐osteoclasts through binding to RANK expressed on the surface of pre‐osteoclasts, which facilitates osteoclastogenesis. OPG, an inhibitor of RANKL, can inhibit RANK‐RANKL interaction.^2^, ^3^ Therefore, it is crucial to maintain the balance between RANKL and OPG for bone remodelling. Previous studies have reported a higher RANKL/OPG (R/O) ratio in gingival crevicular fluid, saliva, blood and gingival tissues of patients with periodontitis compared to that in their healthy tissues, which indicates aggravated periodontitis and active osteoclast formation to accelerate bone loss.^4^, ^5^

Autophagy, particularly macroautophagy, is a survival strategy for maintaining the physiological functions of cells. It is a highly conserved cellular process that can eliminate damaged organelles and facilitate the degradation of misfolded proteins.^6^ A previous study showed that the expression of LC3 is significantly upregulated in periodontal ligament tissues in patients with periodontal disease, compared to that in the healthy patients.^7^ The areas of resorbed alveolar bone also showed higher levels of autophagy in a mouse model of periodontitis.^8^ Moreover, autophagy could facilitate osteoclastogenesis and osteoclastic bone resorption in RAW264.7 cells.^9^ Ubiquitin modification is a crucial step in autophagy.^10^ It has been reported that many proteins in the autophagic process, like TRAF6, Beclin1 and p62 can be modified by ubiquitin.^11^, ^12^ A20, also known as TNFα‐induced protein 3 (TNFAIP3), exhibits an anti‐inflammatory effect. Thus, A20 deficiency in mice triggers inflammation and autoimmune disorders and might eventually lead to death.^13^, ^14^, ^15^, ^16^ Many studies have reported that A20 overexpression in human periodontal ligament cells (hPDLCs) decreases RANKL‐induced osteoclastogenesis in mouse bone marrow‐derived macrophages (BMMs) and that A20 can inhibit autophagy through limiting the K63‐linkage ubiquitination of TRAF6, or by directly deubiquitinating Beclin1.^17^, ^18^

Numerous studies have shown that periodontal tissues are in a hypoxic microenvironment during periodontitis.^19^ The hypoxic condition selected in this study closely resembled that in the microenvironment of periodontitis. hPDLCs are of great importance in the constitution of periodontal tissues. They form a vital link between the cementum and alveolar bone. Similar to osteoblasts, hPDLCs could produce RANKL and OPG and regulate osteoclastogenesis.^20^, ^21^

However, limited research has been performed on whether A20 can regulate osteoclastic differentiation through autophagy in hPDLCs under hypoxic conditions. In this study, we detected the expression of A20 in hypoxia‐stimulated hPDLCs and found that A20 might be a negative regulator of autophagy‐related osteoclastogenesis in periodontitis.

## 2 MATERIALS AND METHODS

### 2.1 Cell culture

hPDLCs were extracted from the middle third of the root surfaces of young patients (12‐16 years old) requiring orthodontic treatment, and informed consent was obtained. The cells were then cultured in α minimum essential medium (α‐MEM) supplemented with 15% fetal bovine serum (FBS, ScienCell, USA) and 1% streptomycin‐penicillin (Gibco, USA) and incubated at 37°C in a humidified atmosphere with 5% CO₂. Upon attaining 90% confluence, hPDLCs were maintained in α‐MEM with 10% FBS and used between passages 3 and 6.

### 2.2 Lentivirus preparation and infection

hPDLCs were transfected using lentivirus A20 purchased from GenePharma (China) for overexpression or silencing studies. After 24 hours transfection, 2.5 µg/mL puromycin was added and the cells cultured for 24 hours to select for positive cells. The infection efficiency was over 80%.

### 2.3 Western blot

Total protein was prepared using cell lysates (Beyotime, China), loaded on 10% or 12% SDS‐PAGE gels and then transferred to polyvinylidene fluoride membranes (Millipore, Germany). Membranes were blocked with 5% non‐fat milk at room temperature for 2 hours and incubated with primary antibodies against GAPDH (1:1000, Proteintech # 10494‐1‐AP), β‐actin (1:1000, Boster # BM0627), A20 (1:1000, Abcam # ab92324), RANKL (1:1000, Abcam # ab9957), OPG (1:1000, Abcam # ab73400), HIF‐1α (1:1000, CST # 36169), LC3B (1:2000, Abcam # ab51520), Beclin1 (1:1000, Proteintech # 11306‐1‐AP), p62 (1:1000, Proteintech # 18420‐1‐AP), ATG5 (1:1000, CST # 12994), TRAF6 (1:1000, Abcam # ab227560), CTSK (1:1000, Abcam # ab187647), TRAP (1:1000, Abcam # ab191406), MMP9 (1:500, Proteintech # 10375‐2‐AP), V‐ATPase D2 (1:1000, Invitrogen # PA5‐44359) overnight at 4°C. These blots were then incubated with secondary antibodies (1:8000, Proteintech # SA00001‐2) or (1:8000, Proteintech # SA00001‐1) for 50 minutes, and the band intensities determined by a chemiluminescent gel imaging system (Tanon, China).

### 2.4 Real‐time quantitative PCR

Total RNA was extracted using TRIzol reagent (Invitrogen, USA) following the manufacturer's protocol. Complementary DNA was synthesized using PrimeScript RT Master Mix, and real‐time PCR was performed using SYBR Premix Ex Taq II (TaKaRa, Japan) at 95°C for 30 seconds, 95°C for 5 seconds and 60°C for 30 seconds for a total of 40 cycles. GAPDH was used as an internal control. The primer sequences were listed in Table 1.

**Table 1:** Primers for Real‐time PCR

Genes	Forward primer	Reverse primer
GAPDH	5′‐GAAGGTGAAGGTCGGAGTC‐3′	5′‐GAGATGGTGATGGGATTTC‐3′
A20	5′‐CTGCTGGCTGCCTGTCTCAAG‐3′	5′‐GTTCTGGAACCTGGACGCTGTG‐3′
RANKL	5′‐TTACCTGTATGCCAACATTTGC‐3′	5′‐TTTGATGCTGGTTTTAGTGACG‐3′
OPG	5′‐TGTGCGAATGCAAGGAAG‐3′	5′‐TGTATTTCGCTCTGGGGTTC‐3′
HIF‐1α	5′‐TCCAAGAAGCCCTAACGTGT‐3′	5′‐ATGATCGTCTGGCTGCTGTA‐3′

### 2.5 Immunofluorescence (IF)

The pre‐treated cells were washed three times with PBS and then fixed with 4% paraformaldehyde, and permeabilized with 0.1% Triton X‐100 (Beyotime, China) for 10 minutes. The cells were then blocked with goat serum for 1 hour, incubated in primary antibody against LC3B (1:2000, Abcam # ab51520) overnight at 4°C, and secondary antibody (1:100, Proteintech # SA00009‐2) for 45 minutes. Nuclei were stained with DAPI (Invitrogen # R37605). Finally, the expression of LC3B was observed by a Leica fluorescence microscope. In osteoclastic assays, 7 μL/mL phalloidin (Cytoskeleton, USA) was used to stain the cytoskeleton.

### 2.6 Transmission electron microscopy (TEM)

Pre‐treated cells were digested and centrifuged into cell clusters. The clusters were fixed with 2.5% glutaraldehyde overnight at 4°C, and then post‐fixed in 1% osmium tetroxide at room temperature for 2 hours. The samples were dehydrated with graded alcohol and embedded in epoxy resin. Next, 50‐nm‐thick sections were excised and stained with uranyl acetate and lead citrate. Autophagosomes were measured by TEM (Tokyo, Japan).

### 2.7 Osteoclast differentiation assay

After treatment under hypoxia (2% O₂) for 24 hours, the conditioned medium (CM) from hPDLCs was collected. Bone marrow mononuclear cells (BMMCs) extracted from 6 weeks old C57BL/6 mice maintained at the Animal Core Facility of Nanjing Medical University. The study was approved by the Ethical Committee of Nanjing Medical University. BMMCs were incubated overnight in α‐MEM with 10% FBS. Floating cells were then collected and further incubated with macrophage colony‐stimulating factor (M‐CSF, 25 ng/mL, PeproTech # 315‐02) for 3 d. Most of the cells became pre‐osteoclasts after treated with 50 ng/mL RANKL (R&D SYSTEMS # 462‐TEC‐010) and 25 ng/mL M‐CSF for 2 days. Half of the medium of pre‐osteoclasts was changed to CM or fresh medium without RANKL and M‐CSF and cultured up to 4 days, for osteoclast differentiation.

### 2.8 Tartrate‐resistant acid phosphatase staining (TRAP staining)

Cells were fixed after incubation and stained for TRAP with a leucocyte acid phosphatase kit (Sigma Aldrich, USA) following the manufacturer's protocol. TRAP‐positive multinucleated (more than 3 nuclei) cells were considered to be mature osteoclasts.

### 2.9 Co‐immunoprecipitation (Co‐IP)

The cell lysates were prepared, and 10 µg of TRAF6 (Santa Cruz Biotechnology # sc‐8409) antibodies and 1 mg of each protein sample were used for co‐IP assays (Thermo Scientific, USA) to detect K48‐specific (1:1000, Abcam # ab140601) and K63‐specific (1:1000, Abcam # ab179434) polyubiquitination. The eluted protein samples analysed by Western blot.

### 2.10 Hydroxyapatite resorption assay

BMMCs were induced into osteoclasts on the surface of fresh bovine cortical bone slices (4 × 4 × 0.2 mm^3^) in 48‐well plates as described previously in osteoclast differentiation assay. After 12 days, the cells on the bone slices were removed. Bone slices were fixed by 4% paraformaldehyde and stained with toluidine blue. ImageJ software was used to measure the areas of resorption pits.

### 2.11 Statistical analysis

All data are presented as the mean ± SD of at least three separate experiments. Statistical analysis was performed by GraphPad Prism 5.0 (GraphPad Software, Inc, San Diego, CA, USA). Statistical comparisons were performed using analysis of variance (ANOVA) and the Student's t test. A value of *P* < .05 was considered as a statistically significant difference.

## 3 RESULTS

### 3.1 A20 is inhibited and the R/O ratio is increased under hypoxia, and the R/O ratio is suppressed by A20

**Figure 1:**
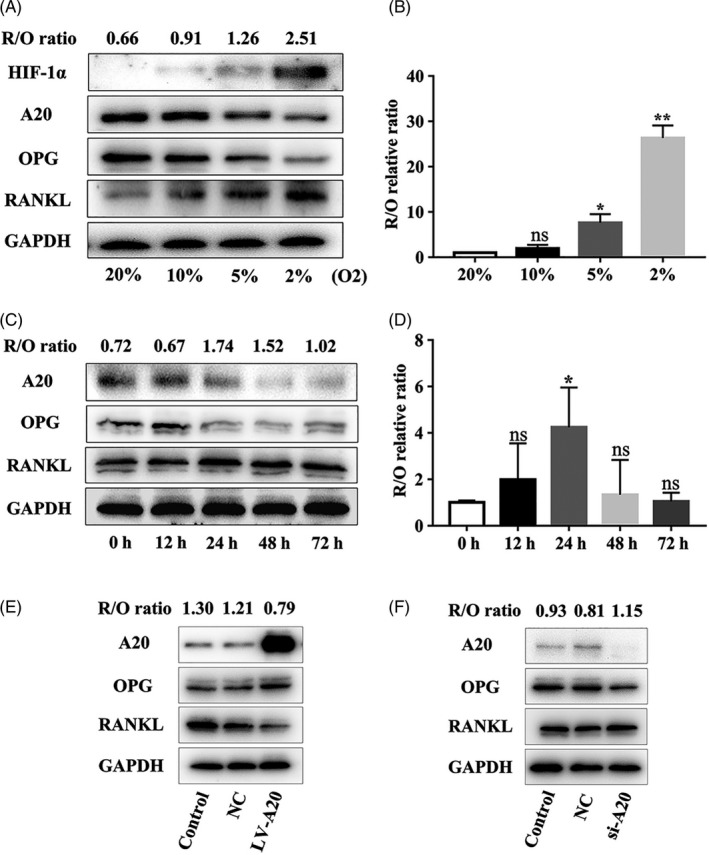
A20 is inhibited and the R/O ratio is increased under hypoxia, and the R/O ratio is suppressed by A20. A, Cells were treated with different oxygen volume fraction for 24 h. The protein expression of HIF‐1α, A20, OPG and RANKL was measured by Western blot, B and the R/O ratio of mRNA expression by RT‐PCR. C, Cells were incubated in 2% O₂ at different time points. A20, OPG and RANKL expression levels were measured by Western blot, D and the R/O ratio of mRNA expression by RT‐PCR. E and F, The protein expression of A20, OPG and RANKL in cells transfected with A20‐targeted lentivirus for overexpression or silencing for 24 h was detected by Western blot. Data are presented as mean ± SD, *P < .05, **P < .01

2% O₂ was adopted as an experimental condition. To determine whether hypoxia regulates A20 and osteoclast‐related factors, hPDLCs were incubated in 20%, 10%, 5%, 2% O₂ for 24 hours. The results showed decreased A20 and OPG protein levels, while the level of RANKL was increased under 2% O₂ compared to other groups (Figure 1A). The maximal R/O ratio of protein and mRNA was observed in 2% O₂ (Figure 1A, B). hPDLCs were then incubated in 2% O₂ for 0, 12, 24, 48 and 72 hours. The results showed decreased protein expression of A20 and OPG, whereas the level of RANKL was enhanced and reached a peak at 24 hours. Moreover, the maximal R/O ratio of protein and mRNA was at 24 hours (Figure 1C, D). Thus, 2% O₂ for 24 hours was chosen as the optimal pro‐osteoclastogenic condition for hPDLCs. These results also demonstrated that A20 was inhibited and R/O ratio was increased by hypoxia.

To confirm the effect of A20 on osteoclast‐related factors, hPDLCs were transfected with lentivirus A20 for 24 hours. The protein expression of RANKL and R/O ratio was significantly decreased, but the expression of OPG was increased in the LV‐A20 group (A20 overexpression) compared to the control and negative control (NC) group (Figure 1E). While A20 silencing had an opposite effect to that of increased RANKL expression and R/O ratio, the expression of OPG was decreased in the si‐A20 group (Figure 1F). These results demonstrated that the R/O ratio was suppressed by A20.

### 3.2 A20 is negatively regulated by autophagy in a time‐dependent manner and can delay the upregulation of R/O ratio

**Figure 2:**
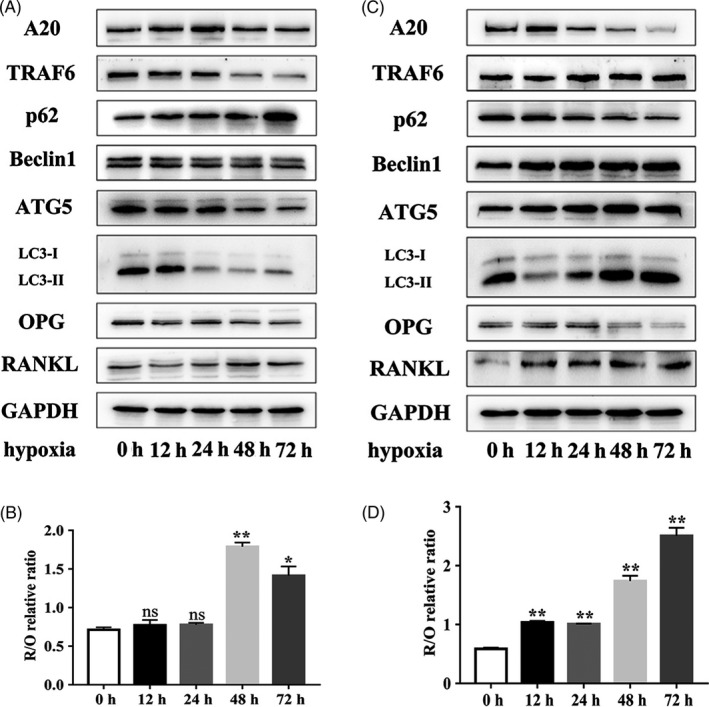
A20 is negatively regulated by autophagy in a time‐dependent manner and can delay the upregulation of R/O ratio. A and B, Cells were transfected with lentivirus A20 for overexpression, followed by treatment in 2% O₂ for 0, 12, 24, 48 and 72 h. The protein expression of A20, TRAF6, ATGs, osteoclast‐related factors and the R/O ratio in the LV‐A20 group was observed by Western blot. C and D, Cells were transfected with lentivirus A20 for silencing, followed by treatment in 2% O₂ at different time points. The protein expression of A20, TRAF6, ATGs, osteoclast‐related factors and the R/O ratio in the si‐A20 group was detected by Western blot. Data are presented as mean ± SD, *P < .05, **P < .01, ***P < .001

To investigate the effect of A20 on autophagic flux and osteoclast‐related factors under hypoxia, hPDLCs transfected with lentivirus targeting A20 were incubated in 2% O₂ for 0, 12, 24, 48 and 72 hours. In the LV‐A20 group, the protein expression of A20 was increased before 48 hours, whereas the protein expression of TRAF6, LC3‐II, Beclin1 and ATG5 was decreased, and p62 expression was increased in a time‐dependent manner (Figure 2A). The above results demonstrated that autophagy was downregulated with increase in A20. The R/O ratio was increased significantly at 48 hours (Figure 2B), which suggested an increase in the pro‐osteoclastogenic activity of hPDLCs.

In the si‐A20 group, the protein level of A20 was increased at 12 hours followed by reduction, while the level of LC3‐II and TRAF6 followed the opposite trend (Figure 2C). Moreover, the expression of ATG5 and Beclin1 was always increased (Figure 2C). These results demonstrated that autophagy was upregulated with decrease in A20. Meanwhile, the R/O ratio was significantly increased at 12 hours (Figure 2D). These observations showed that A20 was negatively regulated by autophagy in a time‐dependent manner and could delay the upregulation of R/O ratio compared to the silencing group in hPDLCs, under hypoxia.

### 3.3 A20 inhibits osteoclastogenesis through downregulating autophagy under hypoxia

**Figure 3:**
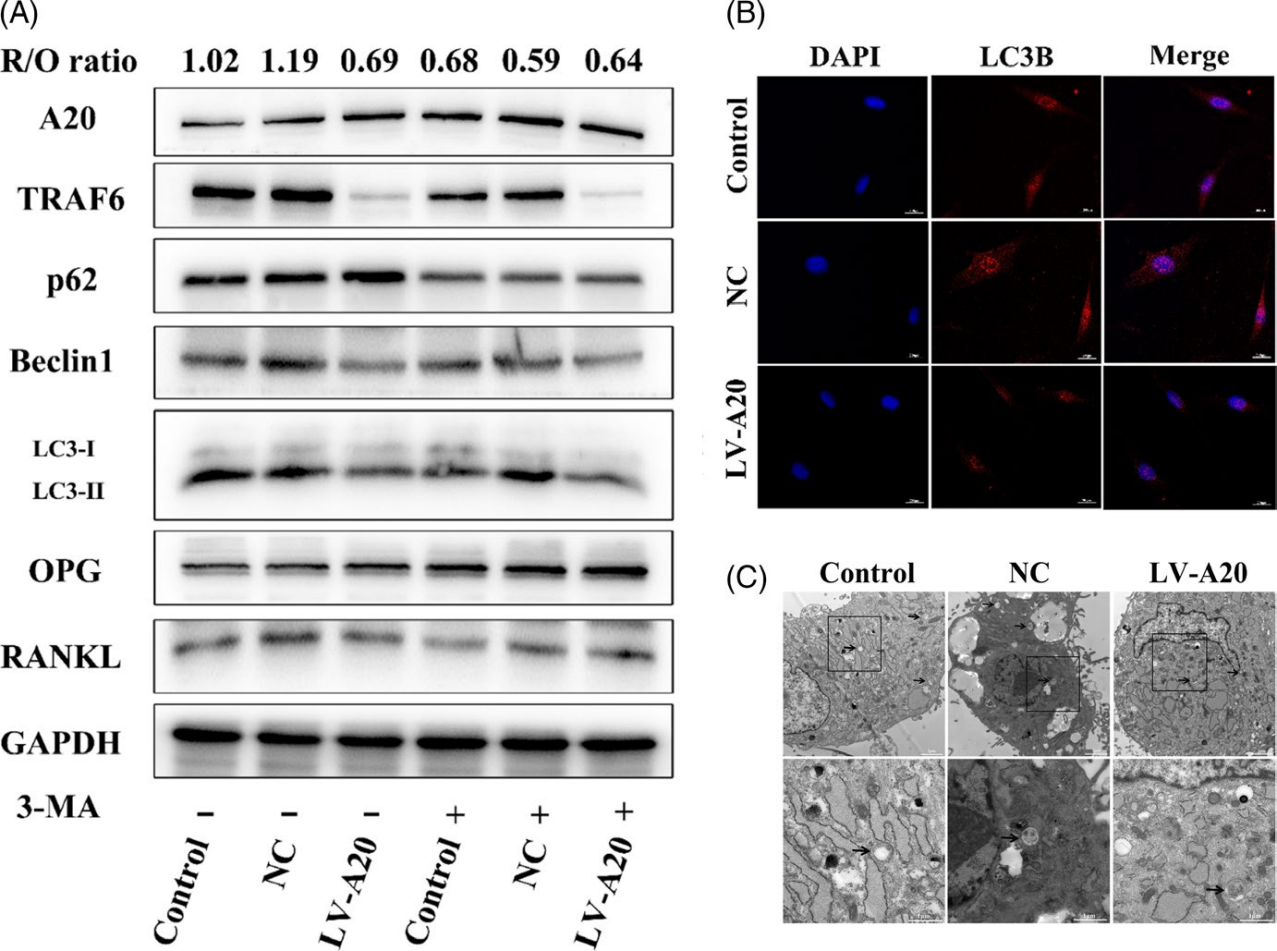
A20 inhibits R/O ratio through downregulating autophagy under hypoxia. A, Cells were transfected with A20‐targeted lentivirus for overexpression and then treated in 2% O₂ for 24 h with or without autophagy inhibitor 3‐MA. The expression of A20, TRAF6, ATGs, and osteoclast‐related factors was detected by Western blot. B, LC3B was measured by IF (bar = 200 μm). C, Autophagosomes were observed by TEM (black arrows, bar = 2 μm and 1 μm)

**Figure 4:**
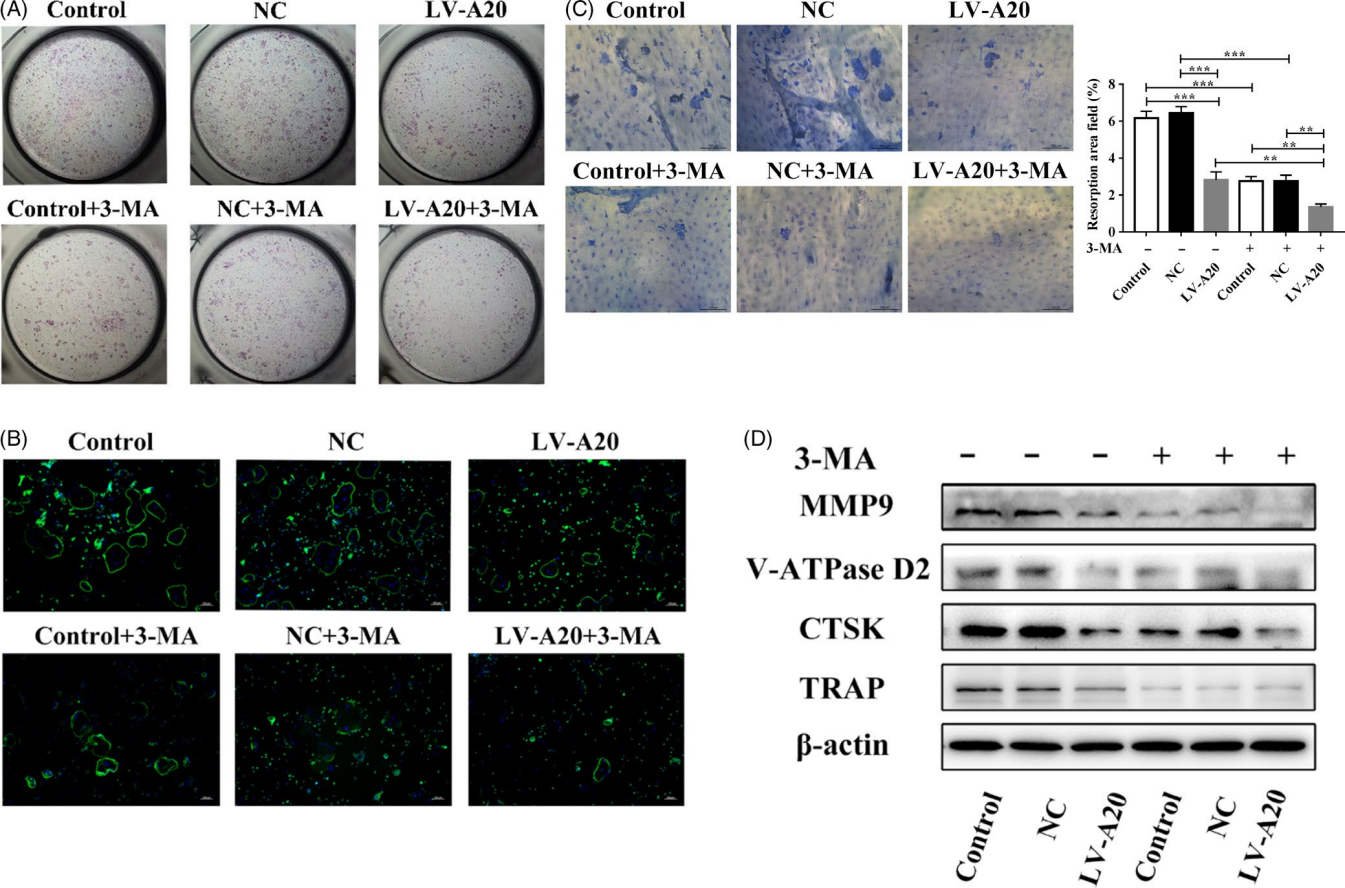
A20 inhibits osteoclastogenesis through downregulating autophagy under hypoxia. A, After becoming pre‐osteoclasts, BMMCs were cultured in CM from cells transfected with lentivirus A20 for overexpression. Osteoclast differentiation was measured by TRAP staining. B, The cytoskeleton of multinuclear cells was detected by IF (bar = 100 μm). C, The areas of hydroxyapatite resorption were measured by toluidine blue staining (bar = 100 μm). D, The expression of osteoclast‐related factors (CTSK, MMP9, V‐ATPase D2 and TRAP) in BMMCs was detected by Western blot. Data are presented as mean ± SD, **P < .01, ***P < .001

To explore whether A20 regulates osteoclast differentiation via autophagy under hypoxia, hPDLCs were transfected with lentivirus A20 for overexpression or silencing. After 24 hours hypoxic treatment without 3‐MA, the protein expression of TRAF6, Beclin1, LC3‐II and R/O ratio was reduced, while the protein expression of p62 increased in the LV‐A20 group (Figure 3A). To further confirm the inhibition of autophagy, the expression of LC3B was observed by IF and the number of autophagosomes was measured by TEM. The results showed weaker fluorescence intensity and fewer autophagosomes in the LV‐A20 group compared to the control and NC group without 3‐MA treatment (Figure 3B, C). The number of osteoclasts and the areas of hydroxyapatite resorption in the LV‐A20 group were decreased (Figure 4A‐C). Meanwhile, the expression of osteoclast‐related factors (CTSK, MMP9, V‐ATPase D2 and TRAP) in BMMCs was decreased (Figure 4D). Through inhibiting autophagy by 3‐MA (5 Mm, MCE, USA), the protein expression of A20 was increased while the R/O ratio was decreased compared with the groups with no 3‐MA treatment (Figure 3A). The number of osteoclasts, the areas of hydroxyapatite resorption and the level of osteoclast‐related factors were decreased (Figure 4A‐D).

**Figure 5:**
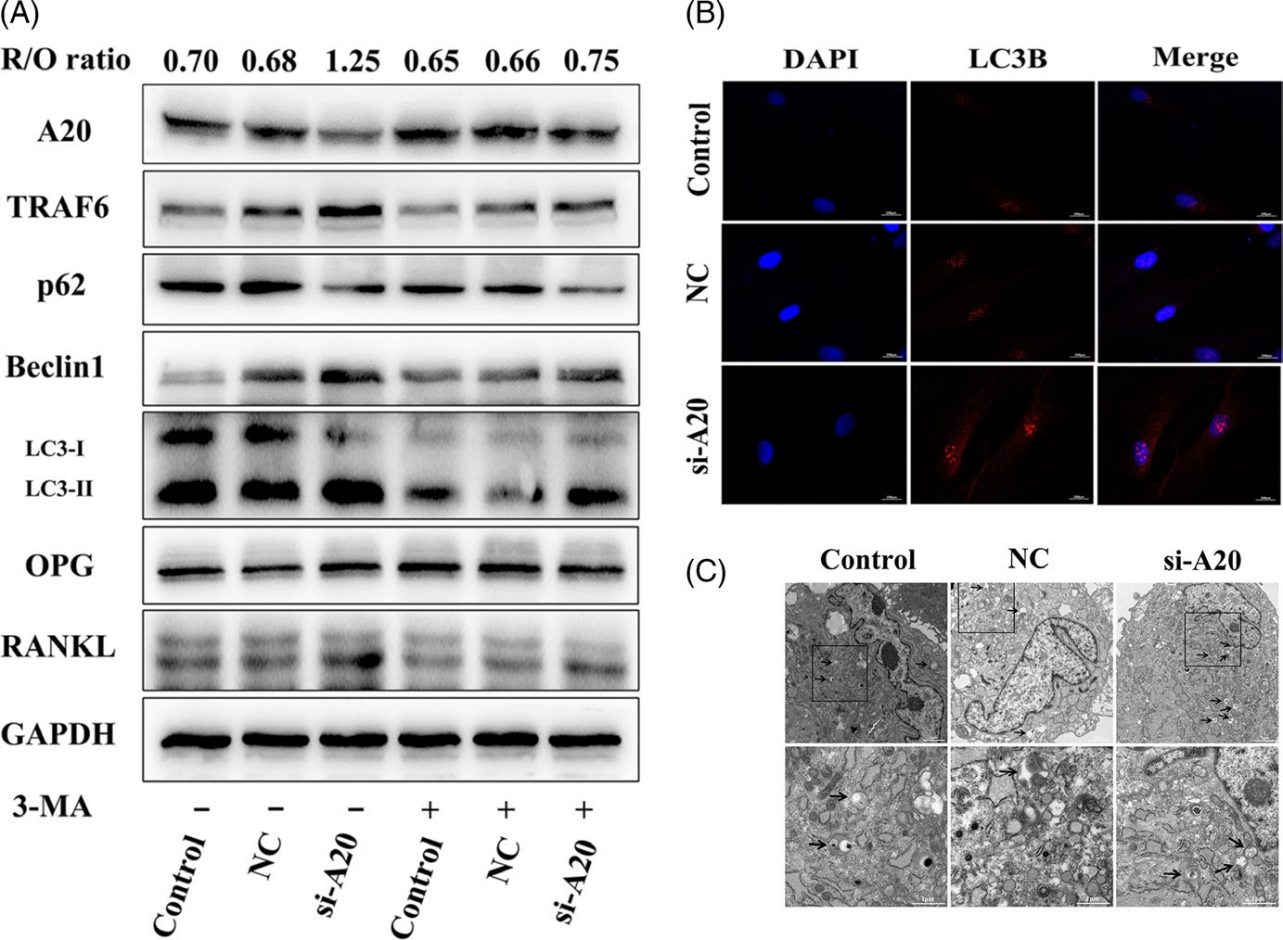
A20 inhibits R/O ratio through downregulating autophagy under hypoxia. A, Cells were transfected A20‐targeted lentivirus for silencing, and then treated in 2% O₂ for 24 h with or without autophagy inhibitor 3‐MA. The expression of A20, TRAF6, ATGs, and osteoclast‐related factors was detected by Western blot. B, LC3B was measured by IF (bar = 200 μm). C, Autophagosomes were observed by TEM (black arrows, bar = 2 μm and 1 μm)

**Figure 6:**
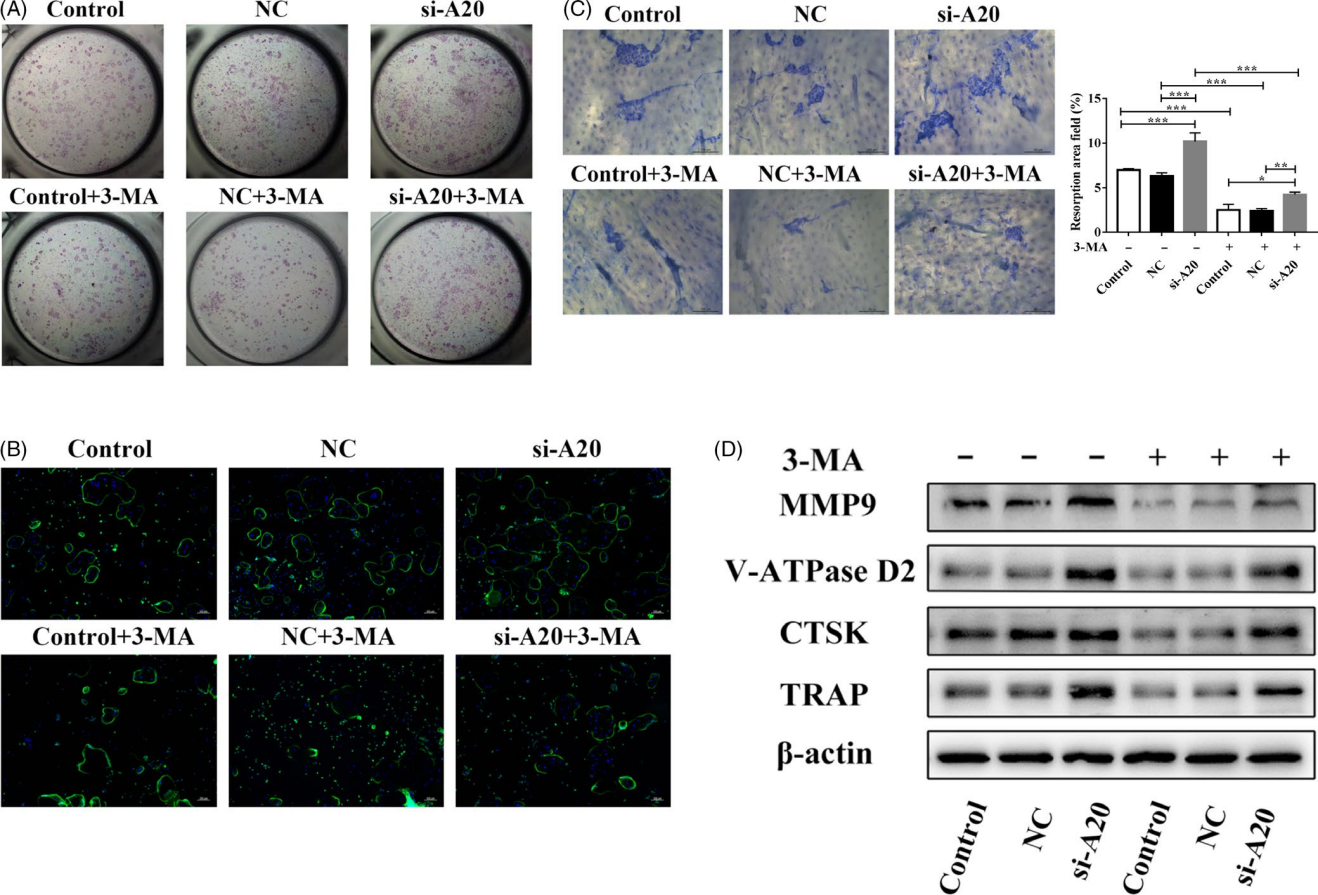
A20 inhibits osteoclastogenesis through downregulating autophagy under hypoxia. A, After becoming pre‐osteoclasts, BMMCs were cultured in CM from cells transfected with lentivirus A20 for silencing. Osteoclast differentiation was measured by TRAP staining. B, The cytoskeleton of multinuclear cells was detected by IF (bar = 100 μm). C, The areas of hydroxyapatite resorption were measured by toluidine blue staining (bar = 100 μm). D, The expression of osteoclast‐related factors (CTSK, MMP9, V‐ATPase D2 and TRAP) in BMMCs was detected by Western blot. Data are presented as mean ± SD, *P < .05, **P < .01, ***P < .001

However, the expression of TRAF6, ATGs (LC3‐II, Beclin1) and R/O ratio were induced in the si‐A20 group compared to the groups without 3‐MA treatment (Figure 5A). In addition, the fluorescence intensity of LC3B was upregulated, and the number of autophagosomes was increased in the si‐A20 group (Figure 5B, C). The number of osteoclasts and the areas of hydroxyapatite resorption were increased in the si‐A20 group, which compared with the control and NC group without 3‐MA treatment (Figure 6A‐C), while the R/O ratio was decreased by 3‐MA (Figure 5A). And, the number of osteoclasts, the areas of hydroxyapatite resorption and the level of osteoclast‐related factors were decreased (Figure 6A‐D). These results indicate that A20 inhibits osteoclast differentiation through downregulating autophagy under hypoxia.

### 3.4 A20 suppresses autophagy dependent the regulation of TRAF6 ubiquitination under hypoxia

**Figure 7:**
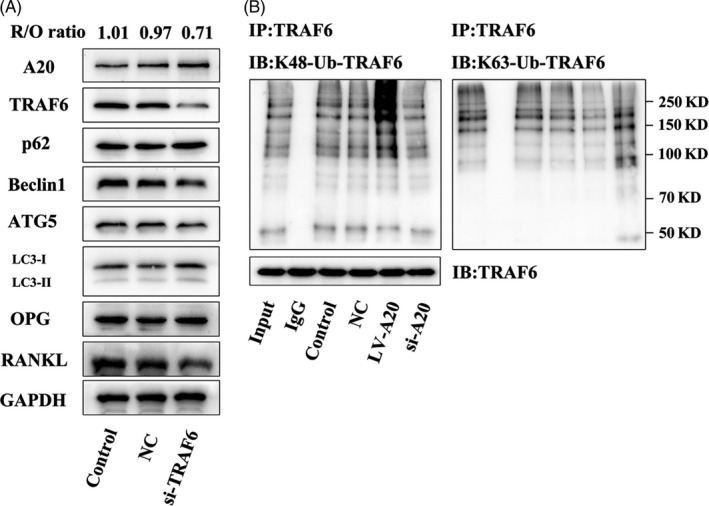
A20 suppresses autophagy dependent the regulation of TRAF6 ubiquitination under hypoxia. A, Cells were transfected with lentivirus TRAF6 for silencing and then treated with 2% O₂ for 24 h. Western blot was applied to detect the protein expression of A20, TRAF6, ATGs and osteoclast‐related factors. B, The co‐IP assay was used to identify different types of ubiquitination (K48‐linked and K63‐linked) on TRAF6

To determine the effect of TRAF6 on A20, hPDLCs were transfected with lentivirus TRAF6 for silencing. In the si‐TRAF6 group, the expression of A20 was increased, autophagy was suppressed, and the R/O ratio was downregulated (Figure 7A). To clarify the precise mechanism of A20 and TRAF6, K48 and K63‐specific polyubiquitin on TRAF6 were detected by co‐immunoprecipitation. In the LV‐A20 group, K48‐specific polyubiquitination was upregulated, and K63‐specific polyubiquitination was downregulated, while K48‐specific polyubiquitination was downregulated and K63‐specific polyubiquitination was upregulated in the si‐A20 group (Figure 7B). It has been reported that K48‐linked polyubiquitin chains promoted the degradation of proteasome, while K63‐linked polyubiquitin chains mediate ubiquitination.^22^ Thus, these results showed that A20 may suppress autophagy through promoting the degradation of TRAF6 and inhibiting the ubiquitination of TRAF6.

## 4 DISCUSSION

Alveolar bone resorption is a vital clinical characteristic of periodontitis. Therefore, investigation of the exact mechanism of the osteoclastic process is particularly urgent for periodontitis therapy. A20 is known to suppress the NF‐κB signalling pathway to exert the anti‐inflammation effects.^15^

*Hong et al* found that A20 overexpression decreased the protein expression of nuclear p65, and the number of osteoclasts induced by LPS and nicotine.^17^ It indicates that A20 overexpression inhibited NF‐κB pathway and osteoclastogenesis. Moreover, TRAF6 is a key factor in NF‐κB signalling and autophagy.^18^ It has been previously reported that A20 inhibits TLR4‐induced autophagy in macrophages.^12^ Therefore, this study was aimed at determining the relationship between A20 and autophagy in anti‐osteoclastogenesis in hPDLCs under hypoxia. In this study, A20 downregulated osteoclast differentiation by limiting autophagy, and this limitation was dependent on the regulation of TRAF6 ubiquitination in hypoxia‐induced hPDLCs. In conclusion, these results demonstrated that A20 exerted an anti‐osteoclastogenic effect through the inhibition of TRAF6‐dependent autophagy in hPDLCs under hypoxic conditions.

A previous study has reported conditioned medium of Ad‐A20 in hPDLCs decreased RANKL‐induced osteoclastogenesis in mouse BMMs.^17^ The study demonstrated that hPDLCs were able to act as osteoclast‐supporting cells to modulate osteoclast differentiation of BMMs. As previous studies have reported, hypoxia could induce osteoclast differentiation in RAW264.7.^23^, ^24^ Our study reveals for the first time the role of A20 in modulating osteoclast differentiation in hPDLCs in hypoxic microenvironment. In other studies, A20 level was always decreased when stimulated by D‐GalN and LPS in liver tissues.^25^ However, in pulmonary artery endothelial cells, the expression of A20 increased rapidly and then decreased under the condition of hypoxia.^26^ This may be explained by the difference in cell types or stimulation mode. It is worth mentioning that we found that despite A20 was overexpressed in hPDLCs, it was still consumed and decreased under the sustained stimulation of hypoxia in this study. It may contribute to the insufficient amount of A20 in anti‐osteoclastogenic effect.

Recent studies have reported that the expression of A20 was increased upon inhibiting autophagy with 3‐MA in Pam3CSK4‐stimulated Atg7 CKO macrophages, compared to that in WT cells.^27^ In other studies, A20 silencing has been found to promote basal LC3 conversion in HeLa cells as well as ATG16L1 expression in the intestinal epithelium.^28^, ^29^ Therefore, we can conclude that A20 may inhibit autophagy. *Lin et al* reported that autophagy facilitated osteoclast differentiation and bone resorption in mouse BMMs in vitro and that conditional knockdown of Atg7 in monocytic cells prevents structural damage in the joints of hTNF‐α mice.^30^ Inhibition of CTSK by siRNA downregulated autophagy and TRAF6 expression to modulate bone destruction.^31^ Meanwhile, *Arai et al* reported that TRAP‐positive osteoclasts in the resorbed alveolar bone around the ligated tooth exhibit higher levels of LC3B in ligature‐induced periodontitis mouse model, which the process was induced by Beclin1.^8^ In addition, *An et al* reported that autophagy‐related proteins, such as LC3B‐II, Beclin1, ATG12 and ATG7, were enhanced in the periodontal ligament tissues in patients with periodontitis.^7^ These results showed that autophagy was activated in periodontitis. In our study, it was proved that A20 inhibited autophagy, reduced TRAF6 expression and decreased the number of TRAP‐positive osteoclasts and the areas of hydroxyapatite resorption. When inhibiting autophagy with 3‐MA, A20 expression was induced, while the expression of TRAF6, the number of osteoclasts and the areas of hydroxyapatite resorption were reduced (Figures 3, 4, 5, 6). Previous studies demonstrated that R/O ratio higher than 1 suggested an increase in osteoclast activity.^5^ On the contrary, the result was opposite.^32^ Our results showed that the overexpression of A20 delayed the increase of R/O ratio, and it demonstrated that A20 overexpression could delay the increase in pro‐osteoclastogenesis activity in hPDLCs and then inhibit osteoclast differentiation. The above findings indicate that A20 could suppress osteoclastogenesis by autophagic inhibition in hPDLCs subjected to hypoxia. TRAF6, as an important adaptor molecule, is involved in the downstream of RANKL/RANK, TLR, IL‐1βR and other classical pathways.^33^ It is a key factor to activate NF‐κB pathway to promote the release of proinflammatory cytokines and osteoclast‐related factors. It is reported that suppression of TRAF6 inhibited the phosphorylation of p38 to decrease the expression of proinflammatory cytokines induced by Porphyromonas gingivalis in hPDLCs.^34^ *Tang et al* have reported that the expression level of TRAF6 in hPDLCs was markedly upregulated upon the activation of Toll‐like receptors and nucleotide‐binding oligomerization domain.^35^ Meanwhile, our research group found that TRAF6 was a major signal molecule for IL‐17‐dependent RANKL regulation in hPDLCs, and TRAF6 interfering inhibited the phosphorylation of IκBα and JNK, and suppressed the IL‐17‐mediated RANKL expression, thus regulate inflammatory bone destruction.^36^

A20‐TRAF6 axis is critical in modulating NF‐κB pathway stimulated by LPS. The silence of A20 restores the expression of TRAF6, which upregulates the activity of NF‐κB under the stimulation of LPS.^37^ By promoting the deubiquitination of TRAF6, the combination of TRAF6 and A20 is interfered and the activation of NF‐κB is inhibited.^38^ This is consistent with the result that A20 expression was increased in TRAF6‐silencing cells (Figure 7A). A previous study reported that A20 plays a role in deubiquitination to cleave the K63‐linkage ubiquitination of TRAF6 or directly affect the K63‐linkage ubiquitination of Beclin1 to inhibit autophagy in macrophages.^12^ In addition, the inhibition of TRAF6 ubiquitin‐ligase activity with PRDX1 led to the downregulation of autophagy activation.^39^ It has been shown that LPS‐induced A20 expression facilitates TRAF6 degradation in human peripheral blood mononuclear cells.^37^ These studies indicate A20‐TRAF6 axis is closely related to autophagy. In addition, A20 decreased the K63‐linkage ubiquitination and increased the K48‐linkage ubiquitination of TRAF6 (Figure 7B). The K63‐linkage ubiquitination facilitates TRAF6 to exert ubiquitin function, while the K48‐linkage ubiquitination is involved in the degradation of TRAF6.^22^ Therefore, this result indicates that A20 could inhibit ubiquitination of TRAF6, and accelerate TRAF6 degradation to suppress autophagy in hPDLCs under hypoxia.

In conclusion, we found that A20 exerted an anti‐osteoclastogenic effect via inhibition of autophagy under hypoxic conditions. Inhibition of autophagy resulted in the regulation of TRAF6 ubiquitylation by A20. Thus, this study demonstrated that A20 inhibits osteoclastogenesis through inhibition of TRAF6‐dependent autophagy in hPDLCs under hypoxic conditions, which may provide A20 as a new therapeutic target for treating bone loss in periodontitis.

## CONFLICT OF INTEREST

The authors declare no conflicts of interest.

## AUTHOR CONTRIBUTIONS

KY, CW and YY designed the experiments; YX, YY and LL were involved in funding acquisition; KY and CW conducted the experiments; WH and XW were involved in methodology design; KY, CW, YY and XW analysed the data; KY and CW prepared the manuscript; YX, LL, YY and SR reviewed and revised the draft. All authors have read and approved the final manuscript.

## ETHICAL APPROVAL

The experiments in this article were approved by the Ethical Committee of Nanjing Medical University (No. 2017‐370).

## Data Availability

The data that support the findings of this study are available from the corresponding author upon reasonable request.
